# Genomic Characterization and Molecular Detection of Rehmannia Allexivirus Virus, a Novel *Allexivirus* Infecting *Rehmannia glutinosa*

**DOI:** 10.3390/microorganisms12050844

**Published:** 2024-04-23

**Authors:** Yanhong Qin, Shuhao Lu, Yi Wen, Shaojian Li, Suxia Gao, Yuxia Liu, Xuemeng Li, Jin Yang, Fengli Wang, Fei Wang, Chuantao Lu

**Affiliations:** Institute of Plant Protection, Henan Academy of Agricultural Sciences, Zhengzhou 450002, China; qinyanhong6040@163.com (Y.Q.); 13629840525@163.com (S.L.); wy412@163.com (Y.W.); lishaojianli@126.com (S.L.); gaosx78@126.com (S.G.); nkyliuys@163.com (Y.L.); meng18790692103@163.com (X.L.); 15738394561@163.com (J.Y.); wangfengli0519@163.com (F.W.); wangfei@hnagri.org.cn (F.W.)

**Keywords:** *Allexivirus*, ReAV, genomic sequence, molecular variation, phylogenetic relationship

## Abstract

*Rehmannia glutinosa* is one of the most important medicinal plants in China and is affected by viral diseases. In this study, a new virus tentatively named Rehmannia Allexivirus virus (ReAV) was identified through high-throughput sequencing, reverse-transcription polymerase chain reaction (RT-PCR), and Sanger sequencing. The complete genome length was 7297 nt and it contained five open reading frames (ORFs) encoding replicase, triple gene block 1(TGB1), TGB2, TGB3, and coat protein (CP). The replicase and CP presented nucleotide homology ranges of 59.9–65.2% and 47.5–55.5% between the nine ReAV isolates and the other 12 species of the genus *Allexivirus*. In the nine isolates, ReAV-20 and ReAV-31 isolates showed breakpoints in the replicase and CP regions, respectively. The other isolates shared 87.2–96.5% nt with the whole genome nucleotide identity. The phylogenetic tree showed that seven ReAV isolates based on replicase, CP, and whole genome sequences were clustered in the same branch and were related to the genus *Allexivirus*. The ReAV detection rates for 60 *R. glutinosa* samples were 73.3–81.7% through RT-PCR using primers targeting the replicase or CP genes. These results demonstrate that ReAV is the dominant virus in *R. glutinosa*. This study provides important evidence for understanding viruses infecting *R. glutinosa* and for establishing efficient strategies to prevent viral spread.

## 1. Introduction

*Rehmannia glutinosa* is a perennial herb belonging to the genus *Rehmannia* in the family *Scrophulariaceae*. It is one of the fifty basic Chinese herbal medicines [1]. *R. glutinosa* is used as fresh or dried root tubers. Fresh *R. glutinosa* has the effects of cooling serum fire and generating fluid to stop thirst, whereas dry *R. glutinosa* clears heat, cools blood, and nourishes yin and blood. Currently, *R. glutinosa* is primarily planted in China, Japan, and Korea [2,3,4]. *R. glutinosa* is mainly produced in Henan, Shanxi, Shandong, Gansu, Shanxi, and Inner Mongolia, of which it is the most famous in Jiaozuo City, Henan Province. *R. glutinosa* has a long history of cultivation spanning more than 1000 years. *R. glutinosa* is cross-pollinated and its seeds cannot be retained. It has long been propagated vegetatively using root tubers during production. Long-term asexual reproduction has also led to serious viral diseases. At present, rehmannia mosaic virus (ReMV) [5], tobacco mosaic virus, cucurbit chlorotic yellows virus (CCYV) [6], tobacco mild green mosaic virus (TMGMV) [7], youcai mosaic virus (YoMV) [8], plantago asiatica mosaic virus [9], tomato mosaic virus, broad bean wilt virus 2 (BBWV2), and columnea latent viroid [10] have been reported to infect *R. glutinosa*. Serious viral diseases are caused by *R. glutinosa* with a yield reduction of more than 60%, and an effective component reduction of 30–50% after the virus infects the plant [11].

Viral particles of the *Alphaflexiviridae* family are curved filaments, usually 12–13 nm in diameter (range: 10–15 nm) [12]. Viruses in this family *Alphaflexiviridae* have a single-stranded positive RNA linear genome of 5.5–9.0 kb in length, with an m7G at the 5′ end and a poly(A) tail at the 3′ end. The family consists of seven genera—*Allexivirus*, *Botrexvirus*, *Lolavirus*, *Mandarivirus*, *Platypuvirus*, *Potexvirus*, and *Sclerodarnavirus*—that include more than 50 species [13]. The family has five to seven open reading frames (ORFs). A large ORF (ORF1) encodes an RNA-dependent RNA polymerase (RdRp or replicase), and ORF2–ORF4 encode putative triple gene block proteins TGB1, TGB2, and TGB3. TGB1 and TGB2 proteins are sufficient for limited cell-to-cell viral movement but TGB3 enhances movement efficiency [14]. ORF5 encodes the putative coat protein (CP) and ORF6 encodes the nucleic acid binding protein (NB) [15]. Some viruses have an additional 42-KDa ORF, such as the vanilla latent virus (VLV) [16]. *Allexivirus* (*Alphaflexiviridae*) was first described in 1970 by Razvjazkina [17]. The genus *Allexivirus* includes 13 recognized virus species (https://ictv.global/report/chapter/alphaflexiviridae/alphaflexiviridae/allexivirus, accessed on 12 March 2024). *Allexivirus* can be divided into two groups based on their genomic organization and host range: *Allium*-infecting allexiviruses (AI) and non-*Allium*-infecting allexiviruses (NAI). The AI group included garlic mite-borne filamentous virus, garlic virus A (GarA), garlic virus B (GarB), garlic virus C (GarC), garlic virus D (GarD), garlic virus E (GarE), garlic virus X (GarX), and shallot virus X (ShVX), eight virus species. They all infect monocotyledons of the genus *Allium*. The major transmission vector of the eight viruses was identified as the eriophyid mite *Aceria tulipae* [18,19]. The NAI group included five species that infect dicotyledonous plants: alfalfa virus S (AVS), *Arachis pintoi* virus (ApV), blackberry virus E (BVE), VLV, and Senna severe yellow virus (SSYMV). Vectors of the NAI group have not yet been identified, and detailed studies on the transmission of these species are required. The genome organization of *Allexivirus* varies among species. The genomes of GarA, GarB, GarC, GarD, GarE, GarX, and ShVX contain seven ORFs, whereas those of AVS, VLV, ApV, and SSYMV contain six ORFs, and BVE contains five ORFs. The AI group encoded a nucleic acid binding protein in the 3′-end-proximal, whereas the NAI group lacked this protein [16,20,21,22,23].

Previous studies have found that field-grown *R. glutinosa* shows obvious symptoms of viral diseases, such as mosaicism, chlorosis, necrotic spots, and distortions [24]. To identify the corresponding *R. glutinosa* viruses, high-throughput sequencing (HTS) and reverse-transcription polymerase chain reaction (RT-PCR) were used for the detection of previously described viruses: YoMV, ReMV, BBWV2, TMGMV, and CCYV [10]. Meanwhile, we identified six contigs partially matching the genome sequences of viruses in the genus *Allexivirus* (*Alphaflexiviridae*), according to the HTS results. The whole genome sequences of the virus were determined by Sanger sequencing. The genome organization, phylogenetic tree, and molecular variations of the virus were analyzed. The newly identified virus was tentatively named Rehmannia Allexivirus virus (ReAV) within the genus *Allexivirus*. Then, the incidence and molecular divergence of this virus was investigated.

## 2. Materials and Methods

### 2.1. Plant Material

Sixty samples of *R. glutinosa*, all showing typical viral symptoms such as mosaic, chlorosis, and distortion, were randomly collected in Wenxian County, Wuzhi County, and Yuzhou City, Henan province, from June to July 2020 (Table 1). A total of 5–6 plants were collected from each field, and 2–3 leaves were collected from each plant and stored at −80 °C.

### 2.2. High-Throughput Sequencing and Data Analysis

A small portion of each collected leaf sample was taken and mixed together as a mixed sample, and sent to Berry Genomics Corporation (Beijing, China) for HTS analysis. First, total RNA was extracted from all leaf samples using an RNAprep Pure Plant Plus kit (TIANGEN Biotech, Beijing, China), and the NEBNext Ultra RNA Library Prep kit for Illumina (NEB, Ipswich, MA, USA) was used to construct a transcriptome library. Sequencing was performed using the Illumina Nova Seq6000 sequencing system (Berry Genomics Corporation, Beijing, China). The processing and analysis of the sequencing data were completed by Wuhan Biowefind Co., Ltd. (Wuhan, China), mainly including the processing and splicing of the sequencing data, BLAST comparison analysis, and annotation of the contigs obtained by splicing.

### 2.3. Amplification of the Full-Length ReAV Genome

According to the detection of 60 *R. glutinosa*, we randomly selected nine isolates to obtain the full sequences of ReAV. Specific primers were designed based on the HTS results (Supplementary Table S1), and primers were synthesized by Sangon Biotech Co., Ltd. (Shanghai, China). Total RNA was extracted using the Column Plant Total RNA Extraction kit (Sangon Biotech Co., Ltd., Shanghai, China). RNA was used as a template to synthesize cDNA using the PrimeScript^TM^ II 1st Strand cDNA Synthesis kit (Takara Biotechnology Co., Ltd., Dalian, China), and the cDNA was stored at −20 °C.

PCR amplification of the cDNA was performed using specific primers. Samples contained: 2× *taq* Master Mix, 10 μL; forward and reverse primers, 0.5 μL each; cDNA, 1 μL; and ddH_2_O added to 20 μL. PCR conditions were 95 °C for 5 min; 35 cycles at 95 °C for 30 s, 55 °C for 30 s, and 72 °C for 30 s, and then a final elongation at 72 °C for 10 min. The PCR product was detected through agarose gel electrophoresis, and the target fragment obtained was recovered and purified using a DNA gel recovery kit. The product recovered was ligated into a pMD19-T vector and transformed into competent *Escherichia coli* TG1 cells. Positive clones (2–4) were selected for sequencing. Sequencing was performed by Sangon Biotech Co., Ltd.

### 2.4. Genome End Sequence Amplification

The SMARTer RACE 5′/3′ kit (Takara) was used to rapidly amplify the cDNA 5′ and 3′ ends, and 5′ rapid amplification of cDNA ends (RACE) reaction was performed according to the manufacturer’s instructions. SMARTer IIA oligonucleotide was added to the 5′ RACE cDNA synthesis reaction. For 3′ RACE, a poly (A) tail was added to the 3′ end of the total RNA using a poly (A) polymerase kit (Takara). Subsequently, the genome end sequence was amplified using nested PCR and the corresponding primers. The product purification, ligation, and transformation steps were the same as those described in Section 2.2. Positive clones (2–4) were selected for sequencing, which was performed by Sangon Biotech Co., Ltd.

### 2.5. Recombination Analysis of Nine ReAV Isolates

The complete genome sequences of nine ReAV isolates were aligned using Clustal X1. The alignment results were analyzed using Recombination Detection Program (RDP) v.4.101 software. The analysis methods included RDP, GENECONV, BootScan, MaxChi, Chimacra, SiScan, and 3Seq. During recombination detection, default parameters were used for each program. Recombination detected in the RDP analysis results using three or more than three methods, and a *p* value for each method of less than 10^–5^, is indicated to be a significant recombination event.

### 2.6. RT-PCR Detection of R. glutinosa Samples

To evaluate the detection rate of ReAV in the 60 samples of *R. glutinosa*, four pairs of primers were designed targeting the replicase and CP viral regions. The primers are shown in Table 2. Total RNA was extracted using a Column Plant Total RNA Extraction kit (Sangon), and reverse transcription using a PrimeScript^TM^ II 1st Strand cDNA Synthesis kit (Takara). RT-PCR was performed as described in Section 2.3. The PCR products were separated using agarose gel electrophoresis; PCR product sizes were verified under ultraviolet lamp with the agarose gel imaging system. The unpurified PCR products were sequenced. Molecular variations were counted after aligning nucleotide sequences using DNAMAN software 7.0. We also evaluated mixed infections of ReAV and YoMV, ReMV, BBWV2, TMGMV, CCYV, and CLVd with *R. glutinosa* in all collected samples (Supplementary Table S2).

### 2.7. Sequence Assembly and ReAV Full Sequence Analysis

The DNAMAN software was used to splice the amplified sequence, and the ORFs of the whole sequence were predicted using the ORF finder on the National Center for Biotechnology Information (NCBI) website (https://www.ncbi.nlm.nih.gov/orffinder/, accessed on 2 July 2023). The full sequence was submitted to GenBank to obtain relevant accession numbers after its genomic structure was examined. The conserved domain database (CDD) on the NCBI website was used to predict the conserved domain in the protein (https://www.ncbi.nlm.nih.gov/Structure/cdd/docs/cdd_search.html/, accessed on 18 October 2023). InterPro (https://www.ebi.ac.uk/interpro/, accessed on 15 November 2023) was used to annotate the non-redundant protein characteristic sequences of protein families, domains, and functional sites. Sequence alignment and homology analysis were performed for the complete ReAV genome, as well as the genes encoded by each functional protein, using DNAMAN software. MEGA7 was used to align the relevant protein sequences, and a bootstrap value of 1000 was used to generate a phylogenetic tree using the neighbor-joining method.

## 3. Results

### 3.1. HTS Data Analysis

HTS is an important tool for identifying known and unknown viruses in plants. In this study, diseased leaf samples were subjected to HTS analysis using the Illumina Nova Seq6000 sequencing system by the Berry Genomics Corporation to obtain 28,522,540 raw reads. After filtration to remove low-quality reads, 27,664,949 high-quality clean reads were obtained for contig assembly. The contigs were subjected to BLAST alignment and annotation. The results show that six known viruses, namely ReMV, BBWV2, YoMV, CCYV, TMGMV, and CLVd, infected *R. glutinosa*. Six viruses were identified through RT-PCR, and TMGMV and CLVd were reported for the first time to infect *R. glutinosa* [10]. In addition, we obtained contigs that did not match the known viruses. Six of these contigs were analyzed through BLASTx in the viral NCBI database. Contig no. 1646, 1237 nt in length, shared 79.71% of amino acids with papaya virus A (QIM41186.1). Contig no. 1290, 1101 nt in length, shared 80.00% of amino acids with garlic virus A (AGC09135.1). Contig no. 636, 1034 nt in length, shared 70.62% of amino acids with papaya virus A (QIM41186.1). Contig no. 387, 1053 nt in length, shared 41.71% of amino acids with garlic virus C (WEX98002.1). Contig no. 249, 6187 nt in length, shared 70.91% of amino acids with the Arachis pintoi virus (YP_009328892.1). Contig no. 648, 1731 nt in length, shared 61.59% of amino acids with garlic virus E (QED44419.1). Based on these results, we hypothesized that this virus may be a new species of *Allexivirus*.

### 3.2. Amplification and Analysis of the Complete Genome Sequence of the New Virus

**Figure 1:**
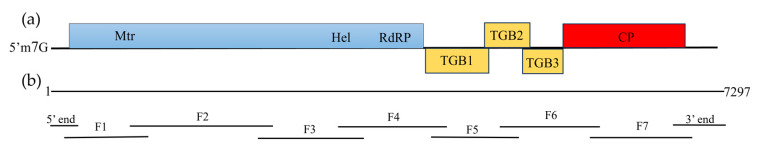
Genome organization of rehmannia allexivirus virus isolate (ReAV-59) showing relative positions of ORFs and their expression products (**a**), and the positions fragments amplified through RT-PCR and 5′ RACE and 3′RACE on six contigs (**b**).

To verify the HTS results and further confirm that the virus was a new species of *Allexivirus*, the complete sequence of one isolate (No. 59) was obtained and characterized. The virus was amplified through RT-PCR, 5′RACE, and 3′RACE for each target fragment (Figure 1). The complete sequence of the virus showed the highest sequence similarity in a BLASTx search with members of the genus *Allexiviruses*. The length of the whole sequence was 7249 nucleotides and contained five computationally predicted ORFs using the ORF finder on the NCBI website, with a poly (A) tail in 3′ terminal. The 5′ and 3′ untranslated regions were 170 nt and 233 nt long, respectively. ORF1 (171–4043 nt, 1289 amino acids) encodes a replicase protein with a predicted molecular mass of 145.90 kDa. The nucleotide position of the methyltransferase motif was 717–759 nt, and that of the helicase motif was 2791–2830 nt using InterPro analysis. The nucleotide position of the RdRp was 2860–3723 nt, using the CDD on the NCBI website. ORF2 (4120–4845 nt, 240 amino acids) encodes TGB1 protein with a predicted molecular mass of 26.50 kDa. ORF3 (4814–5128 nt, 103 amino acids) encodes TGB2 protein with a predicted molecular mass of 11.37 kDa. ORF4 (5022–5321 nt, 98 amino acids) encodes TGB3-like protein with a predicted molecular mass of 10.89 kDa. ORF5 (5309–7063, 583 amino acids) encodes CP with a predicted molecular mass of 63.28 kDa. The nucleotide sequences of the replicase and CP genes of this virus were compared with those of 12 viruses belonging to the genus *Allexivirus*. The results show that the consistency between isolate No.59 and the 12 viruses in *Allexivirus* ranged from 63.8% to 67.2% nt identity for the replicase gene and 24.4–56.8% nt identity for the CP gene. According to the species demarcation criteria, members of the *Allexivirus* share less than 72% nucleotide sequence identity (or 80% amino acid sequence identity) between their CP and replicase, and can be divided into new species. The new virus was tentatively named rehmannia allexivirus virus (ReAV) as a member of a new species in the genus *Allexivirus*.

**Figure 2:**
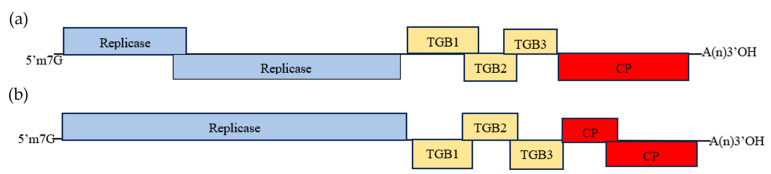
Genome organization of Rehmannia allexivirus virus isolates (ReAV-20 and ReAV-31) showing relative positions of the open reading frames (ORFs) and their expression products ((**a**) ReAV-20, (**b**) ReAV-31).

Based on the acquisition of the complete genome sequence of fifty-nine isolates (PP097219), we also obtained near-full-length sequences of the other eight isolates (ReAV-20, 29, 31, 49, 52, 53, 55, and 58). Genome organization analysis showed that ReAV-29, 49, 52, 53, 55, and 58 isolates (PP097220–PP097225) had the same genomic structure as ReAV-59; they all encoded replicase, TGB1-3, and CP, whereas ReAV-20 (PP097217) and ReAV-31 (PP097218) had a breakpoint in the ORF. The genome organization of the ReAV-20 isolate (Figure 2a) showed that ORF1 (encoding replicase) broke off with the nucleotide positions of 101–1789 nt and 1611–3995 nt. The genome organization of ReAV-31 isolates (Figure 2b) showed that ORF5 (encoding CP) broke off at nucleotide positions of 5302–6217 nt and 5717–7055 nt. To confirm that the breakpoint was not artificially generated because of sequence splicing, we designed primers on both sides of the breakpoint and verified the middle region using RT-PCR and sequencing. The results show a breakpoint in this region.

To clarify the classification status of ReAVs, we conducted a sequence comparison analysis of nine ReAV isolates and twelve other virus species of the genus *Allexivirus* (Table 3). The results show that the whole-genome consistency between the thirteen viruses and the nine ReAV isolates ranged from 48.0% to 55.8%; the replicase had a nucleotide homology range of 59.9–65.2%; and TGB1, TGB2, and TGB3 had nucleotide homology ranges of 39.0–54.5%, 46.4–56.4%, and 30.6–57.0%, respectively. The CP has a nucleotide homology range of 47.5–55.5%. Nine ReAV isolates displayed the highest similarity to the genus *Allexivirus* (*Alphaflexiviridae*) and lower than the species demarcation criteria. So, this virus should be considered a novel species in the genus *Allexivirus*.

### 3.3. Molecular Variation of ReAV Genome Sequences

To clarify the molecular variation of this new virus, the whole genome nucleotide sequences of these isolates were compared and analyzed using the DNAMAN software. The results show that the whole nucleotide sequence consistency of these seven isolates was 87.2–96.5% (Table 4). ReAV-52 and ReAV-29 showed the lowest consistency, whereas ReAV-55 and ReAV-59 showed the highest one. Nucleotide and amino acid sequences of each protein from the seven isolates were analyzed (Table 4). The nucleotide sequence identity of the replicase was 86.7–95.6%, and the amino acid sequence identity was 91.9–98.4%. The nucleotide sequence identity of TGB1 was 85.5–99.6%, and the amino acid sequence identity was 90.5–99.6%. The nucleotide sequence identity of TGB2 was 87.6–99.7%, and the amino acid sequence identity was 94.2–100.0%. The nucleotide sequence identity of TGB3 was 88.0–99.1%, and the amino acid sequence identity was 71.8–100.0%. The nucleotide sequence identity of CP was 86.7–98.3%, and the amino acid sequence identity was 79.2–99.1%. The results show that replicase was more conserved than CP in terms of amino acid sequence identity. TGB3 showed the greatest variation in nt and amino acid levels. 

### 3.4. Recombination Analysis of ReAV Genome

**Figure 3:**
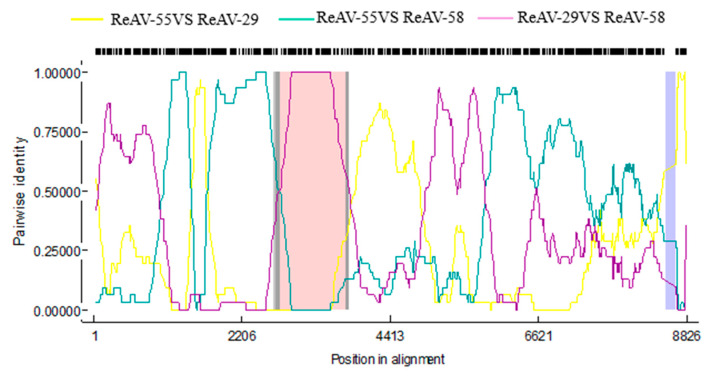
Recombination analysis of ReAV-58 isolate using the recombination detection program RDP4.1. Dark gray regions: 95% break point confidence interval; light gray region: 99% break point confidence interval; purple region: sites excluded from analysis; pink region: tract of sequence with a recombination origin.

Recombination drives virus evolution and new virus production. To explore the virus evolution of nine ReAV isolates, we used RDP4.1 software to have the recombination analysis of nine ReAV isolates. The results show that ReAV-58 recombined with ReAV-55 isolates as the major parent and ReAV-29 isolates as the minor parent. The major recombination may have occurred at position 1–1015 nt, and the minor recombination potentially occurred at position 3477–3593 nt (Figure 3). All seven analysis methods (RDP, GENECONV, BootScan, MaxChi, Chimacra, SiScan, 3Seq) supported this recombination (*p* < 10^−5^), with *p*-values of 1.187 × 10^−22^, 1.682 × 10^−12^, 4.892 × 10^−22^, 7.407 × 10^−10^, 1.736 × 10^−15^, 3.609 × 10^−17^, and 9.325 × 10^−15^, respectively. These results indicate that the obtained ReAV-58 is an ReAV isolate recombined from ReAV-55 isolates and ReAV-29 isolates.

### 3.5. Phylogenetic Analysis of ReAV Isolates and Other Allexivirus Species

**Figure 4:**
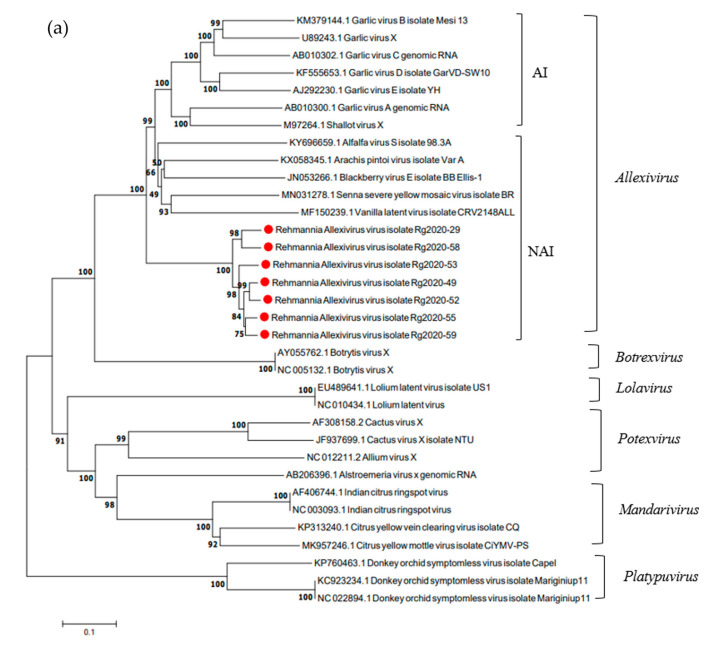

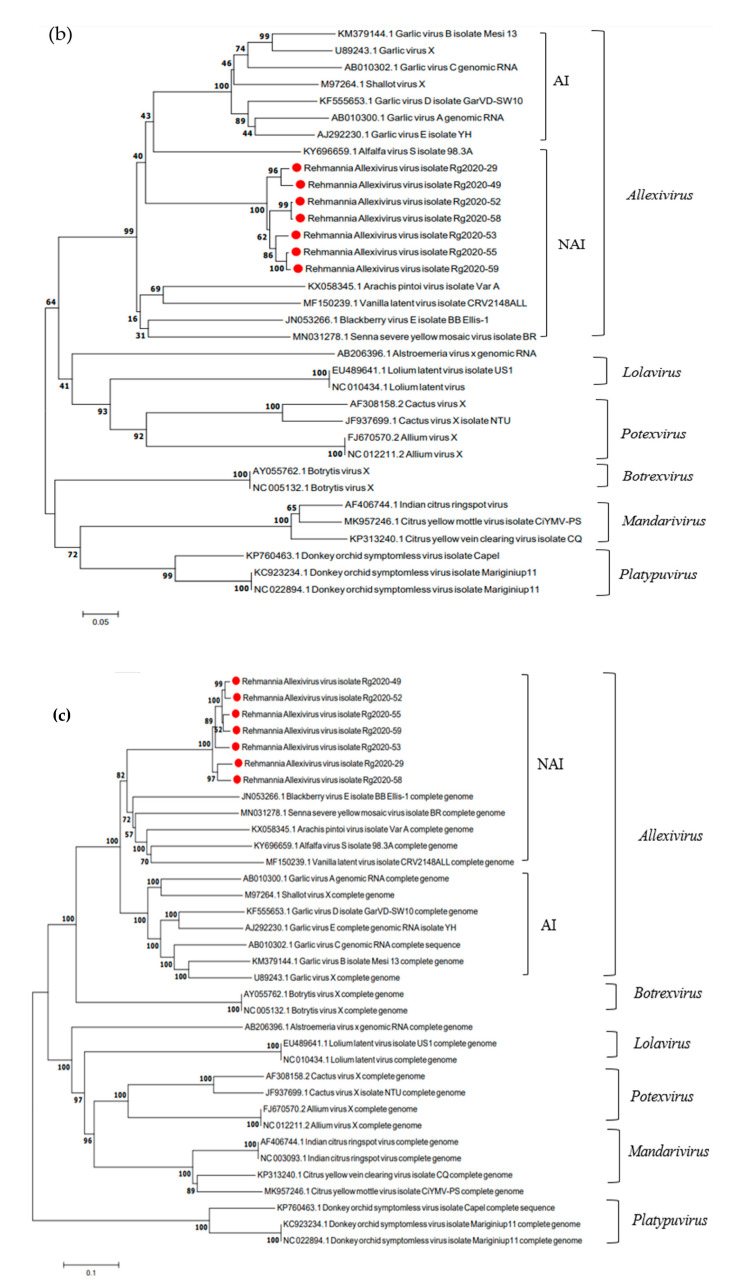
Phylogenetic analysis of ReAV and representative members of the family *Alphaflexiviridae* based on the nucleotide sequences of their replicase (**a**), CP (**b**), and whole genome (**c**). The phylogenetic trees were constructed using a neighbor-joining algorithm with 1000 bootstrap replications. Red dots: Sequences obtained in this study, AI: *Allium*-infecting allexiviruses, NAI: non*-Allium*-infecting allexiviruses. Alfalfa virus S (AVS), *Arachis pintoi* virus (ApV), blackberry virus E (BVE), garlic virus A (GarV-A), garlic virus B (GarV-B), garlic virus C (GarV-C), garlic virus D (GarV-D), garlic virus E (GarV-E), garlic virus X (GarV-X), shallot virus X (ShVX), vanilla latent virus (VLV), Senna severe yellow mosaic virus (SSYMV), *Botrytis* virus X (BotVX), *Lolium* latent virus (LoLV), citrus yellow mottle-associated virus (CiYMaV), citrus yellow vein clearing virus (CYVCV), Indian citrus ringspot virus (ICRSV), donkey orchid symptomless virus (DOSV), *Alstroemeria* virus X (AlsVX), *Allium* virus X (AVX), and *Cactus* virus X (CVX).

To determine the phylogenetic relationship between ReAV and other species in the family *Alphaflexiviridae*, a phylogenetic tree was constructed using MEGA 7.0 software based on whole genome sequences, replicase sequences, and CP nucleotide sequences. These seven ReAV isolates and representative viruses of the family *Alphaflexiviridae* in the NCBI GenBank were subjected to phylogenetic analysis (Figure 4). A phylogenetic tree constructed based on the replicase gene sequences showed that the seven ReAV isolates were on the same branch and were closely related to the vanilla latent virus isolate CRV2148ALL (MF150239.1). The phylogenetic tree constructed based on the CP gene sequences showed that the seven ReAV isolates were also on the same branch and were closely related to alfalfa virus S isolate 98.3A (KY696659.1) and *Arachis pintoi* virus isolate Var A (KX058345.1). According to the phylogenetic tree constructed using whole gene sequences, the seven ReAV isolates were also on the same branch and were closely related to the blackberry virus E isolate BB_Ellis-1 (JN053266.1). In conclusion, phylogenetic tree analysis based on the genome sequence, replicase, and CP genes demonstrated that ReAV had the highest homology with the genus *Allexivirus* and clustered with the non-Allium-infecting *Allexivirus* group.

### 3.6. RT-PCR Detection of ReAV in R. glutinosa Samples

In order to understand the incidence of this new virus in *R. glutinosa*, the leaf samples were collected from 60 *R. glutinosa* plants (Table 1). Four pairs of primers (Table 2) were used to detect ReAV in *R. glutinosa* leaf samples through RT-PCR. The results show that the detection efficiencies of different primer combinations differed. 

**Figure 5:**
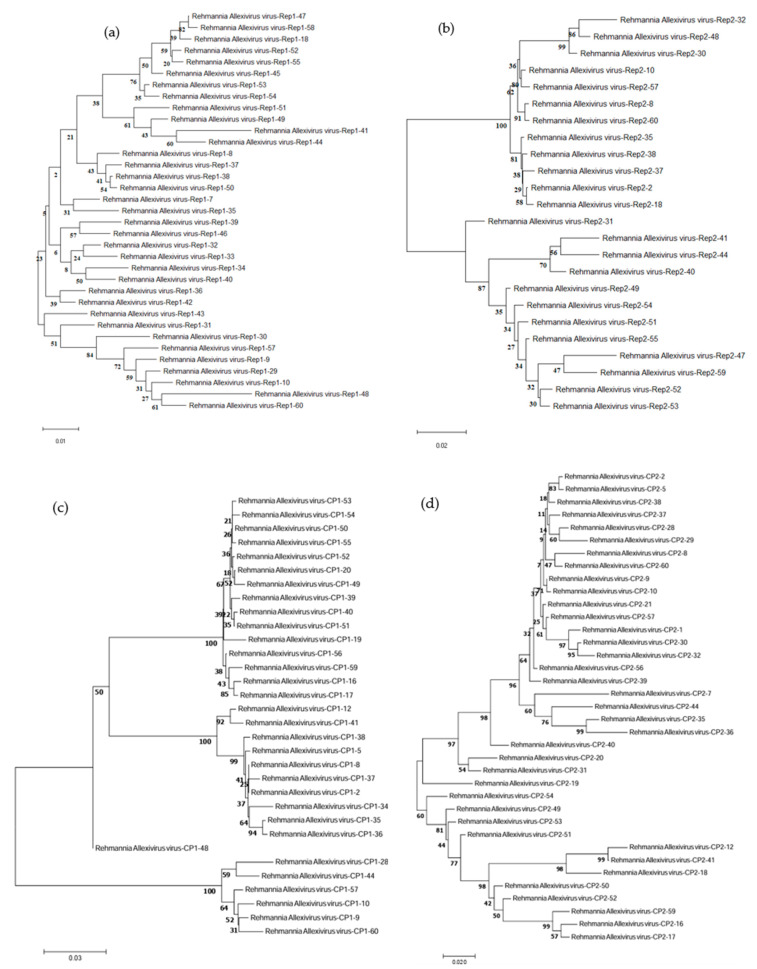
(**a**) Phylogenetic tree based on PCR products of ReAV-rep-1F/1R amplification. (**b**) Phylogenetic tree based on PCR products of ReAV-rep-2F/2R amplification. (**c**) Phylogenetic tree based on PCR products of ReAV-cp-1F/1R amplification. (**d**) Phylogenetic tree based on PCR products of ReAV-cp-2F/2R amplification.

Forty-seven samples were positive for ReAV-rep-1F/1R, with a detection rate of 76.6%. Thirty-five PCR products were randomly selected for sequencing, and the molecular variation was 88.8–99.7%. Sequences were used to construct a phylogenetic tree using MEGA7 (Figure 5a). Regarding molecular variation, ReAV-Rep1-50 and ReAV-Rep1-38 showed the highest consistency, and ReAV-Rep1-50 and ReAV-Rep1-38 were in the same branch of the phylogenetic tree. ReAV-rep-2F/2R detected 44 positive samples with a detection rate of 73.3%. Twenty-four PCR products were randomly selected for sequencing, and the molecular variation was 84.9–99.6%. The phylogenetic tree based on ReAV-rep2 was divided into three branches (Figure 5b), and that based on ReAV-Rep2-31 was divided into a separate branch. It may have had low nt identity with other isolates; ReAV-Rep2-8 and ReAV-Rep2-60 had the highest nt identity. 

Forty-nine samples were positive using ReAV-CP-1F/1R, with a detection rate of 81.7%. Thirty-two PCR products were randomly selected for sequencing, and the molecular variation was 80.7–100%. In the phylogenetic tree (Figure 5c), we found that twenty-five isolates clustered into a branch, ReAV-CP1-48 clustered into a single branch, and the other six isolates clustered into a branch. Forty-nine samples were positive for ReAV-CP-2F/2R, and the detection rate was 81.7%. Thirty-seven PCR products were randomly selected for sequencing, and the molecular variation was 79.3–99.7%. ReAV-CP2-9 and ReAV-CP2-10 showed the highest identity, whereas ReAV-CP2-18 and ReAV-CP2-32 showed the lowest identity with respect to molecular variation. These results were similar in the phylogenetic tree (Figure 5d). In conclusion, the detection rate of ReAV in 60 samples was 96.7% (57/60), and ReAV was the predominant virus infecting *R. glutinosa*.

## 4. Discussion

The occurrence of plant viruses is common in the field, and a variety of viruses has been detected in a variety of crops such as vegetables, grain, and Chinese herbal medicines [25,26,27]. HTS has been widely used for the rapid detection of known or novel viruses infecting plants [28,29,30,31]. We obtained contigs from the host plants using HTS technology. Based on the contigs, we identified all virus-infected plants. Tang et al. detected *Brassica campestris* chinensis cryptic virus 1 (BCCV1) in *Brassica campestris* using HTS [32]. By encoding a conserved RdRp and a putative CP, a homology search and phylogenetic analysis showed that the virus is a new member of the *Deltapartitivirus* genus of the family *Partitiviridae*. Lecoq et al. also used HTS technology to discover the squash chlorotic leaf spot virus, which belongs to the genus *Torradovirus*, in cucurbit crops in Sudan [33]. In the present study, HTS was used to detect suspected viral diseases in *R. glutinosa*. The results show that *R. glutinosa* was infected with multiple viruses. The known viruses are ReMV, BBWV 2, YoMV, TMGMV, CCYV, and CLVd [10]. In addition to the six viruses mentioned, we identified six contigs that matched viruses belonging to the genus *Allexivirus*. According to the PCR amplification and the currently valid criteria proposed by the ICTV *Alphafexiviridae* Study Group [34], the virus in this study is probably a new virus in the genus *Allexivirus*, which we tentatively named ReAV. In addition to these viruses, some contigs can match other viruses belonging to *Caulimovirus*, *Anulavirus*, *Torradovirus*, and *Reoviridae* with low nt identities. These viruses may be new species in the aforementioned genera or families. Further research is needed to acquire complete genome sequences and determine their taxonomic status. Our study demonstrated that viral disease is very serious and intricate in *R. glutinosa* of Henan Province in China.

Mixed viral infections are prevalent in vegetative–reproductive plants. When different types of viruses co-infect a plant, they show a synergistic effect [35,36,37]. Wang et al. found that synergistic interactions occur in mixed infections of lettuce infectious yellows virus (LIYV) and turnip mosaic virus in *Nicotiana benthamiana* plants, resulting in the enhanced accumulation of LIYV [38]. Karyeija et al. identified that sweet potato chlorotic stunt virus can enhance the multiplication of sweet potato feathery mottle virus (SPFMV) in tissues other than the regularly infected ones, perhaps by interfering with the systemic phloem-dependent signaling required in a resistance mechanism directed against SPFMV [39]. Long-term asexual reproduction of *R. glutinosa* leads to serious co-infection with multiple viruses. According to our previous study, six known viruses or viroids infect *R. glutinosa*: YoMV, ReMV, BBWV2, TMGMV, CCYV, and CLVd. Based on the detection results of the 60 *R. glutinosa* samples, the predominant virus species were YoMV, ReMV, BBWV 2, and TMGMV, with detection rates of 100%, 93.3%, 85.0%, and 78.3%, respectively. All samples were co-infected with two or more of these viruses [10]. ReAV, which belongs to the genus *Allexiviruses*, also appears in mixed infections. The AI group is responsible for significant economic impacts. A single infection with either GarV-C or GarV-A decreases garlic bulb weight and diameter by approximately 15% and 5%, respectively [40,41]. A single infection with GarV-D causes a 12% reduction in garlic bulb weight and 7% reduction in bulb quality [42]. Conci et al. found that GarV-A is a mixed infection with *Potyviruses* (onion yellow dwarf virus and leek yellow stripe virus) and *Carlaviruses* (garlic common latent virus). Yield losses are considerably more severe when allexiviruses occur in mixed infections, especially in the presence of *Potyvirus* and *Carlavirus* [43]. In the present study, ReAV, which belongs to the NAI group, was co-infected with *Tobamovirus*, *Fabavirus*, and *Crinivirus*. The interactions between ReAV and six other known viruses belonging to different genera should also be considered. Further studies are required to focus on the decrease in yield and quality caused by mixed infections.

The genus *Allexivirus* includes 13 viruses that can be divided into two groups: AI and NAI. The genome organization of *Allexivirus* varies among species. The AI group contains seven ORFs and the NAI group contains either six ORFs or five ORFs [44]. ReAV contains five ORFs and lacks the 42kDa protein and NABP. ORF1 (replicase) encodes a putative replicase protein with three conserved motifs: methyl transferase, NTPase/helicase, and RdRp [45]. ORF2 (TGB1) encodes helicase, and ORF3 (TGB2) encodes the virus movement domain. TGB1 and TGB2 have been shown to influence viral cell-to-cell movement and systemic transport. ORF4 (TGB3) synthesis requires leaky ribosome scanning initiated by a TGB3 CUG initiator codon [46]. ORF5 (42 KDa) is involved in virion assembly [47]. ORF6 (CP) is a conserved structural core protein. ORF7 (NABP) contains a small cysteine-rich protein (CRP) that acts as a viral transcription factor and silencing suppressor in many viruses [48], but this function was not observed in ShVX [49]. The study found that viral proteins are often multifunctional, and each protein function is important for viral survival and infection. CRP is necessary for the regulation of viral RNA replication, together with pathogenicity determinants, during *Allexivirus* evolution, and to control interactions of the viruses with their plant hosts. The NAI group lacks NABP. The function of this protein may be replaced by that of other proteins, and the functional features of each protein encoded by the NAI group require further study. 

In this study, we obtained full-length or near-full-length gene sequences of nine ReAV isolates. Genomic organization feature analysis revealed a breakpoint in the replicase region of the ReAV-20 isolate and the CP region of the ReAV-31 isolate. We speculate that a single-base mutation may be responsible for this result. Further experiments will be conducted to determine whether this is caused by base mutation. Due to the breaking of the ORF, leading to frameshift translation proteins of the two ReAV isolates (ReAV-20 and ReAV-31), we could not analyze the molecular variation in the other seven ReAV isolates. According to the sequence comparison, we found that two ReAV isolates (ReAV-52, 59) had a low amino acid content consistent with ReAV-29 in the CP gene. Although the amino acid sequence identity of the CP gene was lower than that of the species demarcation criterion (80% amino acid sequence identity), the nucleotide sequence identity was higher than 72%. We considered that the seven ReAV isolates belonged to the same species, and probably separated into two strains or subgroups. The phylogenetic tree based on ReAV replicase, CP, and whole genome sequences was closely related to the vanilla latent virus isolate CRV2148ALL, alfalfa virus S isolate 98.3 A, and blackberry virus E isolate BB_Ellis-1. These three viruses belong to the NAI group; therefore, ReAV should be clustered in the NAI group. Future studies should confirm natural and experimental host ranges.

To understand the detection rate of ReAV in the field, we selected four pairs of specific primers targeting conserved regions of the replicase and CPs to detect 60 samples of *R. glutinosa*. The detection efficiencies of different primer combinations differed. The detection rate was 97.6% in summary, indicating that ReAV is speculated to be the predominant virus infecting *R. glutinosa* and needs to be paid more attention. Future studies should focus on transmission vectors, host ranges, and control methods to limit the spread of this novel virus.

## Figures and Tables

**Table 1:** Sampling information for 60 samples of *Rehmannia glutinosa* suspected of viral disease.

Collection Time	Collection Region	Sample Size
10 June 2020	Wude Town, Wenxian County	18
10 June2020	Dafeng Town, Wuzhi County	15
10 June2020	Xitao Town, Wuzhi County	5
8 July 2020	Xiangyun Town, Wenxian County	5
14 July 2020	Zhangde Town, Yuzhou City	17

**Table 2:** Primers designed for ReAV detection.

Primer Name	Sequence (5′-3′)	Size (nt)
ReAV-Rep-1F	ATGAGCACCCAGCAGGTAGTGAC	507
ReAV-Rep-1R	AAGTGATACGGCTTTGACGGAGA	
ReAV-Rep-2F	CGCCATCGCCCTGTTCAACAAAT	781
ReAV-Rep-2R	AAGCAAGCGGTCGCCCATTCTGT	
ReAV-CP-1F	AGGCTCGCAGTTCAATCAGGTCTTC	624
ReAV-CP-1R	AACTCAGCACATGCCCGTGAGTTT	
ReAV-CP-2F	TACAAACTCACGGGCATGTGCTGA	518
ReAV-CP-2R	TGCAATGTTGCTCCACTATGTCCTTC	

**Table 3:** Percentages of pairwise nucleotide identities between nine ReAV isolates and select *Allexiviruses.*

Virus Name	GenBank No.	Complete Genome	Replicase	TGB1	TGB2	TGB3	CP
garlic virus A	AB010300	49.9–50.9%	61.2–62.1%	48.0–49.9%	51.0–52.6%	36.7–39.5%	50.1–51.7%
garlic virus B	KM379144	51.4–52.4%	60.7–61.7%	48.0–49.7%	50.0–51.0%	**30.6**–32.9%	49.9–51.7%
garlic virus C	AB010302	52.2–-53.0%	61.3–62.5%	50.6–52.2%	51.6–52.6%	38.4–39.8%	47.8–49.1%
garlic virus D	KF555653	51.5–52.2%	60.1–60.9%	49.9–51.3%	49.0–50.0%	35.2–36.1%	50.0–52.2%
garlic virus E	AJ292230	52.8–53.5%	61.2–62.4%	49.0–50.5%	52.9–54.2%	36.1–40.3%	51.3–53.3%
garlic virus X	U89243	49.6–50.9%	59.7–61.1%	49.5–51.2%	48.6–50.8%	36.7–37.7%	51.3–52.2%
Shallot virus X	M97264	**48.0**–49.1%	61.7–62.8%	48.0–49.7%	51.3–53.8%	31.5–35.2%	47.5–49.8%
Alfalfa virus S	KY696659	54.5–55.2%	61.2–61.9%	52.7–**54.5%**	54.5–**56.4%**	48.1–49.1%	50.4–51.1%
Arachis pintoi virus	KX058345	55.1–55.5%	64.5–**65.2%**	48.2–49.7%	49.7–50.6%	33.2–38.4%	50.2–53.3%
Blackberry virus E	JN053266	55.1–**55.8%**	63.3–64.1%	51.7–53.4%	51.9–52.9%	42.1–44.4%	52.7–**55.5%**
Senna severe yellow mosaic virus	MN031278	54.9–55.5%	63.2–64.0%	49.3–50.5%	51.8–53.4%	47.2–**57.0%**	50.1–53.5%
Vanilla latent virus	MF150239	53.6–54.4%	**59.9**–60.8%	**39.0**–41.0%	**46.4**–49.7%	36.1–39.8%	50.2–51.4%

Note: The bold numbers are the maximum and minimum values of the comparison results in the same region.

**Table 4:** Homology of nucleotide and amino acid sequences in the corresponding regions of seven ReAV genome RNAs (%). The percentages for amino acids are shaded. Bold numbers indicate that the ReAV sequence has the highest or lowest consistency with other virus sequences.

Complete Genome							
Virus	ReAV-29	ReAV-49	ReAV-52	ReAV-53	ReAV-55	ReAV-58	ReAV-59
ReAV-29		-	-	-	-	-	-
ReAV-49	88.7%		-	-	-	-	-
ReAV-52	**87.2%**	95.4%		-	-	-	-
ReAV-53	92.4%	90.6%	90.9%		-	-	-
ReAV-55	89.2%	95.0%	93.8%	94.1%		-	-
ReAV-58	91.0%	91.8%	91.6%	90.2%	91.6%		-
ReAV-59	87.4%	94.8%	94.1%	92.6%	**96.5%**	92.8%	
Replicase							
virus	ReAV-29	ReAV-49	ReAV-52	ReAV-53	ReAV-55	ReAV-58	ReAV-59
ReAV-29		92.4%	**91.9%**	97.6%	94.9%	93.9%	92.7%
ReAV-49	**86.7%**		96.7%	93.0%	96.8%	**98.4%**	98.3%
ReAV-52	86.8%	95.5%		92.5%	94.7%	95.4%	96.2%
ReAV-53	92.3%	90.6%	90.3%		95.7%	92.7%	93.4%
ReAV-55	90.5%	95.2%	92.2%	94.1%		96.4%	96.6%
ReAV-58	91.3%	92.4%	88.7%	87.8%	91.4%		98.0%
ReAV-59	87.6%	**95.6%**	93.4%	92.1%	94.9%	94.0%	
TGB1							
virus	ReAV-29	ReAV-49	ReAV-52	ReAV-53	ReAV-55	ReAV-58	ReAV-59
ReAV-29		90.9%	91.3%	97.1%	90.9%	94.2%	**90.5%**
ReAV-49	86.5%		99.6%	93.4%	99.2%	91.7%	98.8%
ReAV-52	86.5%	99.6%		93.8%	**99.6%**	92.1%	99.2%
ReAV-53	97.5%	86.8%	86.8%		93.8%	97.1%	92.9%
ReAV-55	86.5%	99.4%	**99.6%**	86.8%		91.7%	98.8%
ReAV-58	96.0%	86.0%	86.0%	98.1%	86.0%		91.3%
ReAV-59	86.1%	98.3%	98.8%	86.4%	98.3%	**85.5%**	
TGB2							
virus	ReAV-29	ReAV-49	ReAV-52	ReAV-53	ReAV-55	ReAV-58	ReAV-59
ReAV-29		**94.2%**	95.2%	**100.0%**	95.2%	95.2%	96.2%
ReAV-49	87.6%		99.0%	94.2%	99.0%	99.0%	98.1%
ReAV-52	88.3%	98.7%		95.2%	100.0%	100.0%	99.0%
ReAV-53	98.7%	88.9%	89.5%		95.2%	95.2%	96.2%
ReAV-55	**87.6%**	99.0%	99.0%	88.9%		100.0%	99.0%
ReAV-58	87.9%	98.4%	**99.7%**	89.2%	98.7%		99.0%
ReAV-59	87.9%	98.4%	99.0%	89.2%	98.7%	98.7%	
TGB3							
virus	ReAV-29	ReAV-49	ReAV-52	ReAV-53	ReAV-55	ReAV-58	ReAV-59
ReAV-29		87.3%	**71.8%**	81.7%	87.3%	88.7%	88.7%
ReAV-49	**88.0%**		81.7%	71.8%	97.2%	98.6%	98.6%
ReAV-52	87.0%	97.7%		90.1%	81.7%	83.1%	83.1%
ReAV-53	95.8%	88.9%	91.2%		71.8%	73.2%	73.2%
ReAV-55	88.0%	99.1%	97.7%	88.9%		98.6%	98.6%
ReAV-58	88.0%	99.1%	97.7%	88.9%	**99.1%**		**100.0%**
ReAV-59	88.4%	98.6%	97.2%	89.4%	98.6%	98.6%	
Coat protein							
virus	ReAV-29	ReAV-49	ReAV-52	ReAV-53	ReAV-55	ReAV-58	ReAV-59
ReAV-29		88.2%	79.8%	80.3%	80.3**%**	82.7%	**79.2%**
ReAV-49	93.4%		89.0%	89.5%	89.5%	85.9%	87.7%
ReAV-52	87.0%	91.6%		91.4%	91.3%	95.4%	89.4%
ReAV-53	88.5%	91.1%	93.0%		**99.1%**	88.2%	96.2%
ReAV-55	87.1%	90.8%	92.9%	97.9%		88.2%	96.4%
ReAV-58	88.5%	90.5%	98.0%	91.9%	91.8%		86.6%
ReAV-59	**86.7%**	89.9%	92.1%	96.5%	**98.3%**	91.1%	

## Data Availability

Virus clones are available upon request. The raw data generated in this study are available in SRA NCBI (PRJNA1095028).

## Supplementary Materials

The following supporting information can be downloaded at: https://www.mdpi.com/article/10.3390/microorganisms12050844/s1, Table S1. Primers used for the amplification of genome sequences of ReAV-20, 29, 31, 49, 52, 53, 55, 58, and 59 from *Rehmannia glutinosa*. Table S2. Compound infection statistics of five known viruses and four pairs of detection primers (ReAV rep-1F/R, ReAVrep-2F/R, ReAV CP-1F/R, and ReAV CP-2F/R) of ReAV from 60 samples of *Rehmannia glutinosa*.
