# Supplementary material for: Genomic Characterization and Molecular Detection of Rehmannia Allexivirus Virus, a Novel Allexivirus Infecting Rehmannia glutinosa
Source: Microorganisms. 2024 Apr 23;12(5):844. doi: 10.3390/microorganisms12050844 (PMC11123084; doi:10.3390/microorganisms12050844)
Supplement: Supplementary file 1 [file microorganisms-12-00844-s001.zip › Table S1.pdf]

Table S1 Primers used for the amplification of genome sequences of ReAV-20, 29, 31, 49, 52, 53, 55, 58, and 59 from *Rehmannia glutinosa*

fragment	primer	Sequence (5'-3')	Position (nt)	Size (bp)
1	5'RACE	CTAATACGACTCACTATAGGGC	1-22	395
	ReAV-390R	TGCCGTGTCCAGCCAGTTGAG	374-395	
2	ReAV -440F	CACGAACCTAAAGACGTCCT	71-91	1047
	ReAV-1380R	TCGTCTGATTCAAGCCCGTCAC	997-1118	
3	ReAV -1260F	TCGTGGGACAAGCCTCCTACTC	886-907	1608
	ReAV -2670R	GTGTTGACCAGCGTGATGGT	2475-2494	
4	ReAV -2490F	TCCTGCAAATCACAAGAGACGC	2313-2334	1060
	ReAV -3560R	AGTTCGAAATTGAGGAAGGC	3354-3373	
5	ReAV -3300F	CGAGAAAGTCGGGTTCAAGTTC	3107-3128	1204
	ReAV -4500R	GTGTGCGAGGCAAGCTCCTGTG	4290-4311	
6	ReAV -4240F	GGCCATTTCCTCTGACCACAAC	4049-4070	1290
	ReAV -5530R	GCCACGCGCCCAAGCAGGCTAG	5318-5339	
7	ReAV -5340F	TCGTCATTACGGGATCTACCGT	5149-5170	1021
	ReAV -6360R	GCCAACGCGTCTGCGGGCAG	6151-6170	
8	ReAV -6120F	CCACACCATCCAACGGCGGC	5926-5945	1045
	ReAV -7100R	GCTGGCGTGATTCCACGATGGC	6950-6971	
9	ReAV -6610F	GCTACGTCCTCGGCAAGAAGAC	6756-6777	520
	oligodT	------------		
