# Supplementary material for: Genomic Characterization and Molecular Detection of Rehmannia Allexivirus Virus, a Novel Allexivirus Infecting Rehmannia glutinosa
Source: Microorganisms. 2024 Apr 23;12(5):844. doi: 10.3390/microorganisms12050844 (PMC11123084; doi:10.3390/microorganisms12050844)
Supplement: Supplementary file 1 [file microorganisms-12-00844-s001.zip › Table S2.pdf]

Table S2 Compound infection statistics of 5 known viruses and 4 pairs of detection primers (ReAV rep-1F/R, ReAVrep-2F/R, ReAV CP-1F/R, and ReAV CP-2F/R) of ReAV from 60 samples of *Rehmannia glutinosa*

	CCYV	TMGMV	YoMV	ReMV	BBWV2	ReAV rep-1F/R	ReAV rep-2F/R	ReAV CP-1F/R	ReAV CP-2F/R	Number of virus co-infection
1	-	+	+	+	-	-	-	-	+	4
2	+	+	+	+	+	+	+	+	+	6
3	-	+	+	+	+	+	+	-	+	5
4	-	+	+	+	-	+	-	-	+	4
5	+	+	+	+	+	+	+	+	+	6
6	-	+	+	+	+	-	-	-	-	4
7	-	+	+	+	+	+	+	+	+	5
8	-	+	+	+	+	+	+	+	+	5
9	-	-	+	+	+	+	+	+	+	4
10	-	+	+	+	+	+	+	+	+	5
11	-	+	+	+	+	-	-	-	+	5
12	+	+	+	+	+	-	+	+	+	6
13	-	+	+	+	+	+	-	-	+	5
14	+	+	+	+	+	-	-	+	-	6
15	+	-	+	+	+	-	-	-	-	4
16	-	+	+	+	+	+	+	+	+	5
17	-	+	+	+	+	+	+	+	+	5
18	-	+	+	+	+	+	+	+	+	5
19	-	+	+	+	+	+	+	+	+	5
20	-	+	+	+	+	+	+	+	+	5

21	-	+	+	+	+	-	-	+	+	5
22	-	+	+	+	+	+	+	+	+	5
23	-	+	+	+	-	-	-	-	+	4
24	-	-	+	+	-	-	-	+	-	3
25	+	+	+	+	-	-	-	+	-	5
26	+	+	+	+	-	-	-	-	-	4
27	-	+	+	+	+	-	-	+	-	5
28	-	+	+	+	+	+	+	+	+	5
29	-	+	+	+	+	+	+	+	+	5
30	-	-	+	+	+	+	+	+	+	4
31	-	+	+	+	+	+	+	+	+	5
32	+	+	+	+	+	+	+	+	+	6
33	-	+	+	-	-	+	-	-	-	3
34	-	+	+	+	+	+	+	+	+	5
35	-	+	+	+	+	+	+	+	+	5
36	-	+	+	+	+	+	+	+	+	5
37	-	+	+	+	+	+	+	+	+	5
38	-	+	+	+	+	+	+	+	+	5
39	+	-	+	+	+	+	+	+	+	5
40	+	-	+	+	+	+	+	+	+	5
41	+	-	+	+	+	+	+	+	+	5
42	+	-	+	+	+	+	-	+	+	6
43	+	+	+	+	-	+	+	+	-	6
44	+	-	+	+	+	+	+	+	+	5
45	+	-	+	+	+	+	+	-	-	5

46	-	-	+	+	-	+	-	+	-	3
47	-	+	+	+	+	+	+	+	+	5
48	-	-	+	+	+	+	+	+	+	4
49	+	+	+	+	+	+	+	+	+	6
50	-	+	+	+	+	+	+	+	+	5
51	-	+	+	+	+	+	+	+	+	5
52	+	+	+	+	+	+	+	+	+	6
53	-	-	+	+	+	+	+	+	+	4
54	-	+	+	-	+	+	+	+	+	4
55	+	+	+	-	+	+	+	+	+	5
56	+	+	+	-	+	-	+	+	+	5
57	-	+	+	+	+	+	+	+	+	5
58	+	+	+	+	+	+	+	+	+	6
59	+	+	+	+	+	+	+	+	+	6
60	+	+	+	+	+	+	+	+	+	6
Number of positive samples	22	47	60	56	51	47	44	49	49	

Note: "+" represents positive sample, "-" represents positive sample.
